# Developing New Strategies for Relapsed/Refractory Diffuse Large B-Cell Lymphoma

**DOI:** 10.3390/jcm12237376

**Published:** 2023-11-28

**Authors:** Eva Gonzalez Barca

**Affiliations:** Hematology Department, Catalan Institute of Oncology—IDIBELL, University of Barcelona, 08908 Barcelona, Spain; e.gonzalez@iconcologia.net

**Keywords:** diffuse large B-cell lymphoma, relapsed/refractory, therapy, efficacy, toxicity

## Abstract

Diffuse large B-cell lymphoma (DLBCL) is an aggressive and biologically heterogeneous disease. Approximately 40% of patients with DLBCL will experience disease relapse or will be refractory to first-line chemo immunotherapy. In recent years, there have been several new therapeutic agents approved for the treatment of relapsed/refractory (R/R) DLBCL. These agents include anti-CD19 chimeric antigen receptor T-cell (CAR T-cell) and monoclonal antibody therapies such as polatuzumab and tafasitamab. Nevertheless, despite the high efficacy of all these new therapies, there are still patients who do not respond or relapse, representing an unmet clinical need. This review describes new promising therapies that are in clinical development to treat R/R DLBCL.

## 1. Introduction

Diffuse large B-cell lymphoma (DLBCL) is the most common subtype of non-Hodgkin lymphoma. It is a clinically aggressive and biologically heterogeneous disease. Immunochemotherapy with six to eight cycles of rituximab, cyclophosphamide, doxorubicin, vincristine, and prednisone (R-CHOP) has been the standard for first-line treatment for the last 25 years [1], with which more than 60% of patients are cured. Since the early 2000s, different treatment strategies have been tested to improve the results obtained with R-CHOP without success [2,3,4,5]. Nevertheless, in a recent phase III trial, polatuzumab vedotin R-CHP (a modified R-CHOP in which vincristine is replaced by polatuzumab vedotin, an anti-CD79b antibody–drug conjugate) regimen demonstrated an improvement in progression-free survival (PFS) when compared with the standard R-CHOP (76.7% versus 70.2% at 2 years; hazard ratio 0.73) without increasing toxicity, although overall survival (OS) did not significantly differ [6].

**Figure 1:**
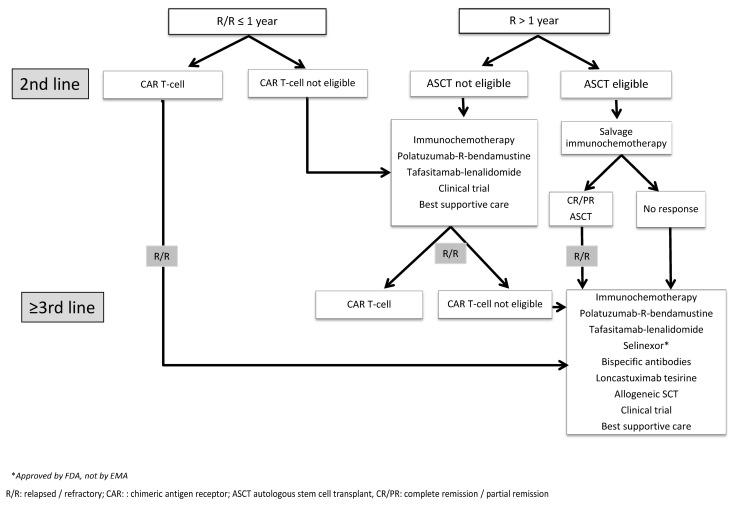
Algorithm for the treatment of relapsed/refractory diffuse large B-cell lymphoma patients.

The standard second-line therapy for relapsed or refractory (R/R) patients to first-line therapy is salvage high-dose chemotherapy, followed by an autologous stem-cell transplant (ASCT) [7] (Figure 1). However, only healthy patients without comorbidities benefit from this strategy [7]. Moreover, as shown in the SCHOLAR-1 study, patients with primary refractory disease or in relapse within 12 months after first-line therapy respond poorly, even with this intensive therapy, with an objective response rate (ORR) of 26%, a complete remission (CR) rate of 7%, and a median overall survival (OS) rate of 6.3 months [8].

Recent novel therapy approaches have changed the treatment landscape for R/R DLBCL patients. Three different constructs of CD19 chimeric antigen receptor T cells (CAR T-cells)—axicabtagene ciloleucel (axi-cel), tisagenlecleucel (tisa-cel), and lisocabtagene maraleucel (liso-cel)—have been approved by the US Food and Drug Administration (FDA) and the European Medicines Agency (EMA) for the treatment of R/R DLBCL following two or more lines of systemic therapy, showing high response rates [9,10,11]. The most up-to-date data for axi-cel demonstrate an OS rate at 5 years of 42.6% without new serious adverse events or deaths after additional follow-ups [12]. CAR T-cells have also been assayed in second-line therapy in high-risk DLBCL patients (refractory or in early relapse during the first 12 months after finishing the first-line treatment) [13,14,15]. In two phase 3 trials, axi-cel and liso-cel showed an improvement in event-free survival (EFS) compared with ASCT and they have been approved by the EMA and FDA [14,15].

An allogeneic stem-cell transplant (allo-SCT) still remains an option for a very small proportion of selected patients (healthy adults without major comorbid conditions) who have failed CAR T-cell therapy and whose disease can be controlled by emerging treatment modalities [16].

Other targeted approaches for R/R DLBCL have been approved in the last years for patients who are poor candidates for ASCT or for CAR T-cell therapy (Figure 1). These include the combination of tafasitamab (an anti-CD19 monoclonal antibody) and lenalidomide [17], the combination of polatuzumab vedotin (an anti-CD79b-conjugated monoclonal antibody) with bendamustine and rituximab [18], and selinexor, an oral inhibitor of exportin 1 (approved by the FDA but not by the EMA) [19]. 

Nevertheless, despite the high efficacy of all these new therapies, there are still patients who do not respond or relapse, representing an unmet clinical need. This review describes some of the new therapies that have been recently approved to treat DLBCL, which, in some countries, are already available or will be available in a short period of time, and also new promising therapies that are under investigation in clinical trials (Table 1).

## 2. Results

### 2.1. Targeted Therapies: Small Molecules

#### 2.1.1. Bruton’s Tyrosine Kinase (BTK) Inhibitors

The B-cell receptor (BCR) signaling pathway is involved in the development of many B-cell malignancies, which makes it one of the targets for inhibitors in B-cell non-Hodgkin Lymphoma (NHL). The constitutive activation of the BCR pathway is implicated in the pathogenesis of the activated B-cell (ABC)–DLBCL subtype, which is associated with NF-κB activation. Drugs targeting this pathway include Bruton’s tyrosine kinase (BTK) inhibitors. BTK inhibitors also have immunomodulatory effects as they favor T-cell differentiation towards the inflammatory Th1 subtype. Four BTK inhibitors have been used for the treatment of lymphomas: ibrutinib, acalabrutinib, zanubrutinib, and pirtobrutinib. Some of these drugs are approved to treat other B-cell lymphoproliferative diseases such as chronic lymphocytic leukemia or mantle cell lymphoma; however, its efficacy in DLBCL is limited. A phase 1/2 trial of single-agent ibrutinib reported an ORR of 25% (CR 10%) and median PFS and OS of 1.6 and 6.4 months, respectively. The results were better for the ABC–DLBCL subtype compared with germinal center B-cell (GCB) DLBCL, with an ORR of 37% versus 5%, a median PFS of 2 versus 1.6 months, and a median OS of 10.3 versus 6.4 months [20]. In a more recent trial, a combination of ibrutinib with other targeted drugs such as regimens with venetoclax, ibrutinib, prednisone, obinutuzumab, and lenalidomide (ViPOR) as well as ViPOR with polatuzumab vedotin had ORRs of 56% (CR 37%) and 64% (CR 36%), respectively [21,22]. An important point is that BTK inhibitors can cross the blood–brain barrier and have demonstrated activity in primary central nervous system (CNS) lymphoma [23]. There are also other trials that continue to evaluate BTK inhibitors in combination with other drugs for DLBCL (such as ibrutinib (NCT02077166), acalabrutinib (NCT04546620), and zanubrutinib (NCT04436107)).

#### 2.1.2. Mucosa-Associated Lymphoid Tissue Lymphoma Translocation Protein 1 (MALT1) Inhibitors

Mucosa-associated lymphoid tissue lymphoma translocation protein 1 (MALT1) is another essential component of the NF-κB pathway. MALT1 inhibition could be a good target for ABC–DLBCL. There are several phase 1 trials that are testing different agents that target MALT1 such as safimaltib and ABBV-525 (NCT03900598 and NCT05618028). However, no clinical data are currently available.

#### 2.1.3. Cereblon E3 Ligase Modulators (CELMoDs)

Immunomodulatory imide drugs (IMIsDs) such as lenalidomide have shown modest activity as a monotherapy for R/R DLBCL. Several next-generation compounds, termed cereblon E3 ligase modulators (CELMoDs), are in development, including avadomide, iberdomide, and golcadomide. The most promising results have been reported with iberdomide. In a phase 1/2 trial, 18 patients were treated with iberdomide with or without rituximab. Among the 7 who received the combination of iberdomide and rituximab, the ORR was 71% (CR 29%). The most common grade 3/4 adverse event was neutropenia in 49% of patients [24]. Golcadomide has been assessed in a phase1 trial in patients with R/R NHL, including 30 with R/R DLBCL, with an ORR of 40% (CR 12%) [25]. These trials are still recruiting patients.

#### 2.1.4. Interleukin-1 Receptor-Associated Kinase 4 (IRAK4) Inhibitors

Interleukin-1 receptor-associated kinase 4 (IRAK4) is a protein that is part of the toll-like receptor pathway that is downstream of MYD88, which enables the activation of the NF-κB and MAPK pathways. Around 40% of ABC–DLBCL patients have MYD88 mutations. Preclinical data suggest that the IRAK4 inhibitor emavusertib results in lymphoma cell death in vitro with MYD88-mutated indolent B-cell lymphomas as a single agent and as a combination partner with BTK or PI3K inhibitors in unselected populations [26]. Emavusertib is now being explored as a monotherapy and in combination with ibrutinib in an ongoing phase 1 trial for different lymphomas (NCT03328078). Preliminary safety and efficacy results for different NHL patients have been reported [27].

### 2.2. Immunotherapy

#### 2.2.1. Conjugated Monoclonal Antibodies

Loncastuximab tesirine is a CD19-directed humanized monoclonal antibody conjugated to a potent pyrrolobenzodiazepine dimer toxin. In a phase 2 LOTIS-2 trial, 145 patients with R/R DLBCL who had a median of 3 previous lines of treatment were included (17% primary refractory; 9% after CAR T-cell failure). Therapy was intravenously administered every 21 days until disease progression or unacceptable toxicity. The ORR was 48% and the CR was 24%, with a median duration of response (DOR) of 13.4 months [28]. Concerns have been expressed that using an anti-CD19 agent may compromise subsequent CAR T-cell therapy. Fourteen patients were identified from the phase 1 and phase 2 trials; they successively received CAR T-cell therapy, achieving an ORR of 50% (CR 43%), similar to those without a previous anti-CD19 therapy [29]. Thirteen patients treated with loncastuximab after CD19-directed CAR T-cell failure had an ORR of 46.2% (CR 15.4%) [30]. The most common adverse effects identified were increases in gamma-glutamyl transferase (40%), neutropenia (40%), thrombocytopenia (33%), fatigue (37%), anemia (26%), and edema (peripheral 20% or pleural 10%). Toxicity concerns were related to the pyrrolobenzodiazepine and dexamethasone used to help mitigate the edemas. New trials using loncastuximab in combination are now recruiting patients. LOTIS-5 is a phase 3 randomized trial for elderly frail patients with R/R DLBCL that compares 8 cycles of R-loncastuximab versus 8 cycles of R-GemOx (NCT04384484); LOTIS-9 is a phase 2 trial with R-loncastuximab for elderly frail patients with previously untreated DLBCL (NCT05144009).

Zilovertamab vedotin (MK-2140) is an antibody–drug conjugate comprising a monoclonal antibody that recognizes extracellular receptor tyrosine kinase-like orphan receptor 1 (ROR1), a cleavable linker, and the anti-microtubule cytotoxin monomethyl auristatin E. ROR1 is a fetal protein that is highly expressed during early embryonic development, but expressed at very low levels in adult tissues. A pathologic expression of ROR1 is seen in some hematologic and solid tumor cancers. In a phase 1 trial, in which patients with NHL were included, 17 had DLBCL with a median of 4 prior lines of therapy; 71% had received prior CAR T-cells or CAR natural killer (NK) cells and 47% had a GCB–DLBCL subtype. The most frequent grade 3/4 treatment-related adverse events were neutropenia (32%) and thrombocytopenia (11%). The ORR was 29% (CR 18%) for patients with DLBCL [31]. There is an ongoing phase 2 trial for R/R DLBCL using zilovertamab until disease progression, unacceptable toxicity, or withdrawal (WAVELINE-004). So far, 40 patients have been enrolled; 24 (60%) with ≥3 prior lines of therapy, 25% with a prior ASCT, and 28% with failed prior CAR T-cell therapy. The current ORR is 30%. The most common grade 3/4 toxicities are neutropenia (18%) and anemia (15%). Treatment-related peripheral neuropathy has occurred in 15% of patients and none were ≥grade 3 [32]. There are different trials ongoing in combination with other drugs for R/R DLBCL (NCT05139017) and even for first-line DLBCL (NCT05406401).

Brentuximab vedotin is an anti-CD30 antibody–drug conjugate with the anti-microtubule cytotoxin monomethyl auristatin E. It is indicated for the treatment of Hodgkin lymphoma and CD30+ T-cell lymphomas. About 15–25% of DLBCL patients express CD30; therefore, trials have been performed in this setting. In a phase 2 trial, 49 patients with R/R DLBCL CD30+ (defined as >1% expression) were treated with brentuximab. The majority of patients were refractory to the first-line (76%) and most recent therapies (82%). The ORR was 44% (CR 17%), the median DOR was 16.6 months, and the median PFS was 4 months. It is important to note that the degree of CD30 expression did not correlate with the responses. Adverse events were consistent with known toxicities [33]. In a phase 1 study, a combination of brentuximab and lenalidomide was used in 37 patients, achieving an ORR of 57% (CR 35%); the median PFS and OS were 10.2 and 14.3 months, respectively [34]. Enrollment in a phase 3 trial, named ECHELON-3, using R-lenalidomide with or without brentuximab is ongoing (NCT04404283). 

#### 2.2.2. Checkpoint Inhibitors

##### T-Cell Checkpoint Inhibitors

The immunological interactions between programmed cell death receptor 1 (PD1) and its ligands (PDL1 and PDL2) have been shown to prevent T-cell activation and proliferation, weakening the immune response. An overexpression of other checkpoint receptors such as cytotoxic T-lymphocyte antigen 4 (CTLA-4) has also been proven to lessen immune surveillance in tumors. Antibodies targeting PD1 and CTLA-4 have been developed with the aim of restoring T-cell functions and eventually halting tumor proliferation.

Several checkpoint inhibitors have been studied in R/R DLBCL. The phase 2 KEYNOTE-170 study demonstrated effective antitumor activity and an acceptable safety of pembrolizumab 200 mg administered every 3 weeks for up to 35 cycles (~2 years) in 53 patients with R/R primary mediastinal B-cell lymphoma (PMBCL) whose disease progressed after or who were ineligible for ASCT. In the final analysis, with a median follow-up of 48.7 months, the ORR was 41.5% (CR 21%), the 4-year PFS was 33.0%, and the 4-year OS was 45%. The most common adverse events were neutropenia, asthenia, and hypothyroidism [35]. Several clinical trials are ongoing, using combinations of T-cell checkpoint inhibitors with other drugs such as brentuximab or acalabrutinib [36,37].

##### Macrophage Checkpoint Inhibitors

CD47, which is found in both healthy and malignant cells, regulates macrophage-mediated phagocytosis by sending a “don’t eat me” signal to the signal regulatory protein alpha (SIRPα) receptor. Increasing evidence has demonstrated that blocking the CD47 interaction with SIRPα can enhance cancer cell clearance by macrophages. Several macrophage checkpoint agents are in development, including magrolimab, lemzoparlimab, and evorpacept. In a phase 1b trial, a total of 22 patients (15 with DLBCL and 7 with follicular lymphoma) were treated with magrolimab and rituximab. Patients had received a median of 4 previous therapies and 95% were refractory to rituximab. Among the patients with DLBCL, the ORR and CR rates were 40% and 33%, respectively. The most frequent grade 3/4 adverse event was transient anemia [38]. In a phase 2 trial, 33 patients with R/R DLBCL with a median of 2 prior therapies were treated with magrolimab combined with R-GemOx. After a median follow-up of 11.3 months, the ORR and CR rates were 51.5% and 39.4%, respectively. The median DOR was 18.0 months. The most common grade 3/4 toxicities were hematological [39]. Several trials with other CD47 blockers are ongoing (such as NCT03934814 and NCT03013218).

#### 2.2.3. Bispecific Antibodies

Bispecific antibodies (BsAbs) are a new class of commercially available immunotherapies with clear efficacy in B-cell lymphomas, including in patients who relapse after CAR T-cell therapy. BsAbs combine two monospecific antigen-binding regions from different antibodies into a single antibody molecule with bispecific antigen binding.

##### T-Cell Engagers

Currently, the majority of BsAbs under development are T-cell engagers. They target CD20 in B cells and engage CD3 in T cells. Those with more advanced development in DLBCL are glofitamab and epcoritamab; their use has recently been approved by the FDA and EMA in adult patients with R/R DLBCL following 2 or more lines of systemic therapy.

Glofitamab is a full-length BsAb with a 2:1 configuration, with bivalency for CD20 in B cells and monovalency for CD3 in T cells. In a pivotal phase 2 trial [34], 154 patients with R/R DLBCL were treated with a fixed-duration intravenous glofitamab monotherapy (12 cycles in total). The median number of previous lines of therapy for these patients was 3; 90 (58%) were primary refractory and 46 (30%) had failed CAR T-cell therapy. At a median follow-up of 12.6 months, 39% achieved CR. The results were consistent among the 52 patients who had previously received CAR T-cell therapy (35% of whom achieved CR). The 12-month PFS was 37%. Discontinuation of glofitamab due to adverse events occurred in 9% of the patients. The most common adverse event was cytokine release syndrome (CRS) in 63% of the patients (grade 3 or higher CRS in 4%). Grade 3 or higher neurologic events appeared in 3% of patients [40].

Epcoritamab is a full-length IgG1 BsAb targeting CD3 and CD20, which is subcutaneously administered in 28-day cycles until disease progression or unacceptable toxicity. In a pivotal phase 2 trial, 157 patients were included [41]. The median number of prior lines of therapy was 3; 96 (61%) had primary refractory disease and 61 (39%) had failed prior CAR T-cell therapy. At a median follow-up of 10.7 months, the ORR was 63.1% and the CR was 38.9%. The response rates were similar across key pre-specified subgroups. The median duration of response was 12.0 months. The most common adverse events were CRS in 50% of the patients (grade 3 in 2.5%), pyrexia in 24%, and fatigue in 23%. Immune effector cell-associated neurotoxicity syndrome occurred in 6.4%.

Another BsAb in development for R/R DLBCL is odronextamab, a fully human IgG4-based BsAb targeting CD20 and CD3. In a phase 1 trial [42], 145 patients with R/R NHL were enrolled (94 to a dose escalation and 51 to a dose expansion). The median number of previous therapies was 3; 42 (29%) patients had received previous CAR T-cell therapy and 119 (82%) were refractory to the last line of therapy. The recommended dose for expansion in patients with DLBCL was 160 mg. The most common grade 3 or worse adverse events were anemia (25%), lymphopenia (19%), neutropenia (19%), and thrombocytopenia (14%). CRS occurred in 41 (28%) patients. In 15 patients with DLBCL without previous CAR T-cell therapy who received doses of 80 mg or higher, the ORR was 53% (all CR); among the 30 patients with previous CAR T-cell therapy who had received doses of 80 mg or higher, the ORR was 33% and the CR was 27%.

There are data that confirm that BsAbs can safely and effectively be combined with other therapies and many clinical trials are now recruiting patients. As some examples, there is a phase 3 trial comparing glofitamab in combination with rituximab and gemcitabine and oxaliplatin (R-GemOx) with R-GemOx in R/R DLBCL patients not eligible for ASCT (NCT04408638). Glofitamab has also been combined with R-CHOP in first-line DLBCL and compared with polatuzumab R-CHP (NCT03467373). There is also a trial of epcoritamab in combination with different chemotherapies for patients with R/R DLBCL using rituximab, cytarabine, dexamethasone, and oxaliplatin/carboplatin (R-DHAX/C) for patients eligible for ASCT and GemOx for patients not eligible for ASCT as well as other regimens (NCT04663347). There is also a phase 3 trial ongoing with the combination of first-line epcoritamab-R-CHOP compared with R-CHOP (NCT05578976). 

##### Natural Killer (NK)-Cell Engagers

There is a new category of BsAbs targeting NK cells under investigation. NK-cell engagers are not subject to major histocompatibility complex (MHC) restrictions that could lead to a lower toxicity. CD16 is the most common NK target. There is a phase 1 clinical trial ongoing using the anti-CD30-CD16 molecule AFM13 to treat CD30-positive lymphomas, including some DLBCLs (NCT04074746). Only the CD16A isoform is able to activate tumor cell destruction, but CD16A is easily lost due to cleavage by ADAM17. Different strategies have been developed to avoid this. One option could be the addition of an ADAM17 inhibitor in combination with CD16. Another option that is under investigation is to target multiple NK receptors such as NKp30, NKp46, NKG2D, and DNAM1. Some preclinical results targeting CD16 and CDp46 in NK cells and CD19 in tumor cells have already been reported [43].

##### Other Types of BsAbs

Other types of BsAbs are in early development. These are BsAbs targeting multiple immune checkpoint receptors (such as PDL1-41BB, PD1-LAG3, and PDL1- CTLA-4) or multiple antigens in the tumor cell (such as CD47- CD19 and CD19-CD22). They have been used in monotherapies as well as in combination with other therapies.

#### 2.2.4. New CAR Strategies

The high rate of relapse in up to 60% of DLBCL patients after CAR T-cell therapy represents a major challenge. On the other hand, the safety of CAR T-cell treatment, with the risk of CRS and/or immune effector cell-associated neurotoxicity syndrome (ICANS), is still a concern. Therefore, there is ongoing extensive research to improve the efficacy and toxicity of these products.

##### Bispecific/Trispecific/Universal CAR T-Cells

One of the mechanisms for relapse after CAR T-cell treatment is tumor cell antigen escape. This can occur through the downregulation of the target antigen by the malignant cells or due to tumor heterogeneity in the antigen expression. One of the proposed solutions is the use of dual- or triple-target CAR T-cells, or even the development of universal CAR T-cells, which would allow a single line of CAR T-cells to bind to several antigens by using different adaptor molecules as ligands [44]. 

Data regarding a CD19-CD20 bispecific CAR T-cell construct in a dose escalation and expansion study have been published. In total, 22 patients (11 with R/R DLBCL) with a median of 4 previous lines of therapy were infused. Grade 3/4 CRS occurred in 1 (5%) patient and grade 3/4 neurotoxicity in 3 (14%) patients. The ORR to the dose of 2.5 × 10^6^ cells per kg with non-cryopreserved infusion (*n* = 12) was 100% (CR 92%). The researchers noted that a loss of CD19 was not seen in relapsed patients or treatment failures [45]. Other similar trials for bispecific CAR T-cell products are underway (NCT04007029 and NCT04215016)

##### ON-Switch and OFF-Switch CARs

After infusion, CAR T-cells set off a chain of immunological reactions that lead to the killing of the lymphoma cells, but also to immunological toxicities. The ON-Switch CAR separates the signaling domain from the costimulatory domain, and T cells can be activated by the addition of small molecules that promote the assembly of the two fragmented CARs. The extent of cell activation can be determined by the dosage of the molecules. The OFF-switch CAR, also called small molecule-assisted shutoff (SMASh)-CAR, contains a degron domain in the CAR structure that allows degradation of the CAR when protease inhibitors are administered to the patient, thereby downregulating T-cell activity [46,47].

ON and OFF CAR T-cells using the clinically approved drug lenalidomide have been developed. Degron tags with enhanced sensitivity to lenalidomide-induced degradation have been identified and used to generate OFF-switch-degradable CARs. To create an ON-switch, a lenalidomide-inducible dimerization system was engineered that required both lenalidomide and the target antigen for activation. Subtherapeutic lenalidomide concentrations controlled the effector functions of ON- and OFF-switch CAR T-cells [48]. 

##### Combinations of CARs with Other Immunostimulatory Drugs

One of the mechanisms of CAR T-cell failure is the presence of an immunosuppressive tumor microenvironment. This is being addressed by combining CAR T-cell therapy with other drugs.

One strategy has been a combination with immune checkpoint drugs. A study looked at pembrolizumab administration in R/R patients after treatment with CD19 CAR T-cell therapy for 12 patients, 11 with DLBCL. The median number of prior therapies was 4, the median PFS after CAR T-cell infusion was 2.2 months, and the median time to the first pembrolizumab dose was 3.3 months. The most relevant adverse grade 3/4 events after pembrolizumab were neutropenia in 3 patients and CRS in 1. Other grade 1/2 toxicities included an infusion reaction in 1 patient and fever in 2. In total, 11 patients were evaluable for response; the ORR after pembrolizumab was 27% (1 CR and 2 PR) and 9/12 patients showed a re-expansion peak in peripheral blood CAR T-cells [49]. Following these results, some of the new CAR constructs are seeking to create a similar effect by including a PD1-blocking molecule in the construct. In the ongoing ZUMA-6 trial, R/R DLBCL patients are treated with an anti-CD19 CAR T-cell regimen, followed by 4 doses of atezolizumab (1200 mg/dose) as an IV infusion every 21 days (NCT02926833).

Another strategy has been the combination of CAR cells with BsAbs. There are trials ongoing in which BsAbs are given to patients who do not achieve CR at day 30 after CAR T-cell infusion (NCT04889716, NCT04703686, and NCT05633615). The results have not been reported so far.

##### Off-the-Shelf CAR Cells: CAR–NK

Several factors make access to CAR T-cells difficult for many patients. One of these factors is the long waiting time required for the production of personalized T cells. To overcome this, universal allogeneic CAR T-cells and other CARs with alternative effector cells (off-the-shelf CARs) are currently being developed.

As mentioned above, NKs can identify target cells without MHC restrictions and do not cause graft-versus-host disease (GVHD). Therefore, they are a potential option for the production of off-the-shelf CARs. NK cells can be obtained from different sources such as allogeneic pluripotent stem cells or umbilical cord blood.

In a phase 1/2 trial, HLA-mismatched anti-CD19 CAR–NK cells derived from cord blood were administered to 11 patients with relapsed or refractory CD19-positive lymphoid malignancies. The administration of CAR–NK cells was not associated with the development of CRS, ICANS, or GVHD. The maximum tolerated dose was not reached. In total, 8 (73%) patients responded and 7 (64%) had CR. Responses were rapid and seen within 30 days after infusion, and the infused CAR–NK cells expanded and persisted at low levels for at least 12 months [50]. Several CAR–NK-based phase 1 clinical trials are now recruiting patients with different lymphomas (NCT05487651 and NCT05336409).

## 3. Discussion

Fortunately, we have a much larger armamentarium than a few years ago to treat DLBCL patients. Among all these new therapies, the most mature data reported are with the BsAbs glofitamab and epcoritamab, and with the conjugated anti-CD19 monoclonal antibody loncastuximab. In the pivotal trials of the BsAbs, more than 150 extremely high-risk patients were included, many of them in progression after CAR T-cell therapy, achieving CR rates of around 40%. Although these results are excellent, it should not be forgotten that the follow-up was very short; nevertheless, the PFS at 1 year with glofitamab was 37% and the PFS at 6 months with epcoritamab was 44% [40,41]. Regarding loncastuximab, 145 very high-risk R/R DLBCL patients were treated in the pivotal trial; the ORR was close to 50% and the CR rate was around 25%. Again, this trial had a short follow-up; the PFS at 6 months was around 45% [28]. However, due to these results, these compounds have recently been approved by the FDA and EMA to treat R/R DLBCL after two or more lines of systemic therapy. 

Among the other compounds discussed, those that seem most promising are CELMoD iberdomide combined with rituximab [24] and CD47 inhibitors such as magrolimab [38]. Few patients have been treated with these drugs, but the high ORR preludes further development. On the other hand, drugs already approved in other indications such as the conjugated anti-CD30 antibody brentuximab [33,34] and the T-cell checkpoint inhibitor pembrolizumab [35] appear to be very effective in certain subtypes of DLBCL.

Immunotherapy with CAR T-cells has emerged as a very effective therapy for DLBCL, but not all patients respond and many of them progress. Different strategies are under investigation to improve the efficacy and to reduce the toxicity of these products. One of the strategies is the use of dual-target CAR T-cells that could reduce the risk of tumor cell antigen escape. In a small group of patients treated with a CD19-CD20 bispecific CAR T-cell product, the ORR was 100% and the toxicity was lower than with other CARs [45]. As a result of these preliminary results, trials are currently being carried out that include a larger number of patients. Another important problem of CAR cells is that they have to be engineered for each individual patient and the manufacturing process is long and expensive. The manufacturing of universal CARs using allogeneic NK cells as effector cells is ongoing. In a preliminary trial, the response rate was very high without CRS or ICANS toxicity [50]. For this reason, there are different phase 1 trials underway.

In summary, new therapies have been recently approved and will presently be available for DLBCL patients and there are many others in development. Nevertheless, there are still many questions to be answered. As we have more therapeutic options, we have to assess how we will sequence them. A comparison among different strategies is difficult as there are no trials that directly compare them and the characteristics of the patients differ among the different trials. Therefore, the optimal setting and sequencing of therapies is unknown. Another important point is how these new therapies can be combined to improve efficacy without increasing toxicity. It is also important to select the best treatment for each individual patient. One of the options to select the best treatment modality is to identify the predictive biomarkers of treatment responses and toxicities. Hopefully, the results of the ongoing trials will help us to answer some of these questions.

## Figures and Tables

**Table 1:** New therapies in clinical development for relapsed/refractory diffuse large B-cell lymphoma.

Therapy	Type of Therapy	Mechanism of Action	Molecules
Targeted therapy	Small molecules	BCRi	Ibrutinib
			Acalabrutinib
			Zanubrutinib
		MALT1i	Safimaltib
			ABBV-525
		CELMoDs	Avadomide
			Iberdomide
			Golcadomide
		IRAK4i	Emavusertib
Immunotherapy	Conjugated MoAb	Anti-CD19 + pyrrolobenzodiazepine	Loncastuximab tesirine
		Anti-ROR1 + monomethyl auristatin E	Zilovertamab vedotin
		Anti-CD30 + monomethyl auristatin E	Brentuximab vedotin
	Checkpoint inhibitors	T cell: PD1-PDL1	Pembrolizumab
		Macrophage: CD47-SIRPα	Magrolimab
	Bispecific Ab	T-cell engagers CD20-CD3	
			Glofitamab
			Epcoritamab
			Odronextamab
		NK-cell engagers CD30-CD16	
			AFM 13
	CAR cells	Bispecific/trispecific/universal CAR T-cells	
		ON/OFF-switch CARs	
		Allogeneic CAR NK cells	

BCRi: Bruton’s tyrosine kinase inhibitor; MALT1i: mucosa-associated lymphoid tissue lymphoma translocation protein 1 inhibitor; CELMoDs: cereblon E3 ligase modulators; IRAK4i: interleukin-1 receptor-associated kinase 4 inhibitor; MoAb: monoclonal antibody; ROR1: tyrosine kinase-like orphan receptor 1; PD1-PDL1: programmed cell death receptor 1–programmed cell death ligand receptor 1; SIRPα: signal regulatory protein alpha receptor; NK: natural killer; CAR: chimeric antigen receptor.
